# Removing hepatitis C antibody testing for Australian blood donations: A cost‐effectiveness analysis

**DOI:** 10.1111/vox.13429

**Published:** 2023-05-14

**Authors:** Qinglu Cheng, Veronica C. Hoad, Avijoy Roy Choudhury, Clive R. Seed, Peter Bentley, Sophy T. F. Shih, Jisoo A. Kwon, Richard T. Gray, Virginia Wiseman

**Affiliations:** ^1^ Kirby Institute UNSW Sydney Sydney Australia; ^2^ Australian Red Cross Lifeblood Perth Western Australia Australia; ^3^ UWA Medical School The University of Western Australia Perth Western Australia Australia; ^4^ Department of Global Health and Development London School of Hygiene & Tropical Medicine London UK

**Keywords:** blood donation testing, blood safety, cost effectiveness, hepatitis C

## Abstract

**Background and Objectives:**

The risk of transfusion‐transmitted hepatitis C virus (HCV) infections is extremely low in Australia. This study aims to conduct a cost‐effectiveness analysis of different testing strategies for HCV infection in blood donations.

**Materials and Methods:**

The four testing strategies evaluated in this study were universal testing with both HCV antibody (anti‐HCV) and nucleic acid testing (NAT); anti‐HCV and NAT for first‐time donations and NAT only for repeat donations; anti‐HCV and NAT for transfusible component donations and NAT only for plasma for further manufacture; and universal testing with NAT only. A decision‐analytical model was developed to assess the cost‐effectiveness of alternative HCV testing strategies. Sensitivity analysis and threshold analysis were conducted to account for data uncertainty.

**Results:**

The number of potential transfusion‐transmitted cases of acute hepatitis C and chronic hepatitis C was approximately zero in all four strategies. Universal testing with NAT only was the most cost‐effective strategy due to the lowest testing cost. The threshold analysis showed that for the current practice to be cost‐effective, the residual risks of other testing strategies would have to be at least 1 HCV infection in 2424 donations, which is over 60,000 times the baseline residual risk (1 in 151 million donations).

**Conclusion:**

The screening strategy for HCV in blood donations currently implemented in Australia is not cost‐effective compared with targeted testing or universal testing with NAT only. Partial or total removal of anti‐HCV testing would bring significant cost savings without compromising blood recipient safety.

**Highlights**

- In Australia, the risk of transfusion‐transmitted hepatitis C virus (HCV) infections is extremely low.
- Anti‐HCV testing in addition to HCV RNA nucleic acid testing for screening blood donations does not prevent any additional morbidity in recipients but uses significant resources.
- Partial or total removal of anti‐HCV testing would bring significant cost savings without compromising blood recipient safety.

## INTRODUCTION

Hepatitis C virus (HCV) is a bloodborne virus of global public health concern. In Australia, the main transmission route is the sharing of needles among people who inject drugs. Globally, transmission via the reuse or inadequate sterilization of medical equipment and the transfusion of unscreened blood and blood products are important considerations. Australian Red Cross Lifeblood (Lifeblood) is responsible for the collection and distribution of blood and blood products in Australia. Collections are made from voluntary non‐remunerated donors in Australia. HCV antibody (anti‐HCV) testing is currently used in parallel with HCV RNA nucleic acid testing (NAT) to detect current or past HCV infections. With approximately 1.6 million blood donations collected annually [1], no transfusion‐transmitted HCV infections have occurred with current testing [2].

A previous international modelling study demonstrated that the additional blood safety provided by anti‐HCV testing is minimal with universal NAT [3]. However, universal donor testing with both anti‐HCV testing and NAT continues not only in Australia but also in other developed countries [4, 5]. The current screening strategy is considered as a ‘belt and braces approach’ primarily to mitigate the remote risks of test failure. While the rate of HCV infection in first‐time donors is higher than repeat donors [6], approximately 90% of Australian blood donations are contributed by repeat donors [1]. In addition, plasma for further manufacture collections for plasma‐derived medicinal products has been steadily increasing and now outnumbers whole blood collections. The fractionation process includes pathogen reduction steps that substantially reduce the HCV residual risk, so anti‐HCV testing in this context is unlikely to provide any clinically relevant safety benefit.

The shift in the management of chronic hepatitis C provides further justification for reconsidering HCV donation testing strategies. The use of dual testing was adopted at a time when direct‐acting antiviral (DAA) therapy was not available, and the majority of those infected by HCV would progress to chronic hepatitis C. In the past, testing donors for HCV reduced the incidence of severe conditions such as liver failure, as well as avoiding significant costs associated with managing these conditions [7]. With the advent of DAA, which has demonstrated a cure rate of over 95% [8], in the vast majority of cases, diagnosed HCV infection can be cured.

Given the context of finite healthcare resources and the limited incremental risk–benefit contributed by anti‐HCV testing, a targeted anti‐HCV testing strategy or no anti‐HCV testing may be favourable over current testing. Removing or targeting anti‐HCV will lower the total costs of HCV screening for blood donations, but how this will affect the residual risk of HCV infection and the long‐term costs and health outcomes requires investigation. Cost‐effectiveness analysis is such a tool that can incorporate all available evidence and assist decision‐making in the transfusion medicine [9, 10]. Previous studies have investigated the cost‐effectiveness of adding NAT to anti‐HCV testing in blood donation [11, 12, 13], but the cost‐effectiveness of removing anti‐HCV testing or applying anti‐HCV testing dependent on donor risks, remains unknown. Therefore, this study aims to conduct a cost‐effectiveness analysis of different testing strategies for HCV infection in Australian blood donations.

## MATERIALS AND METHODS

### Alternative HCV testing strategies

In this study, four HCV testing strategies were proposed for comparison:

1. Universal testing with both anti‐HCV and NAT (status quo).
2. Anti‐HCV and NAT for first‐time donors and NAT for remaining donations.
3. Anti‐HCV and individual donor‐NAT (ID‐NAT) for transfusible component donations and pools of 16 donations (MP16‐NAT) for plasma for further manufacture.
4. Universal testing with NAT only.

Under strategies 1, 2 and 4, ID‐NAT is used for transfusible component donations and MP16‐NAT for plasma for further manufacture.

### Decision‐analytical modelling

**FIGURE 1:**
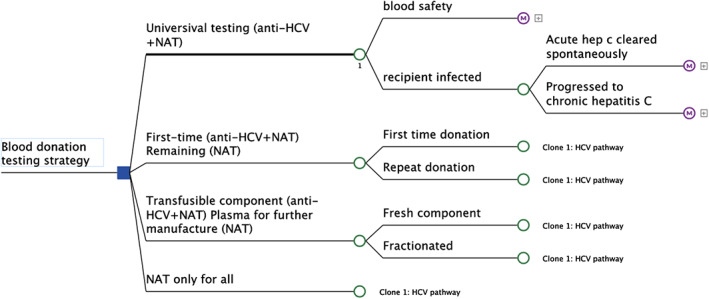
Decision tree model structure for hepatitis C virus (HCV) testing in blood donations. Anti‐HCV, hepatitis C virus antibody; NAT, nucleic acid testing.

**FIGURE 2:**
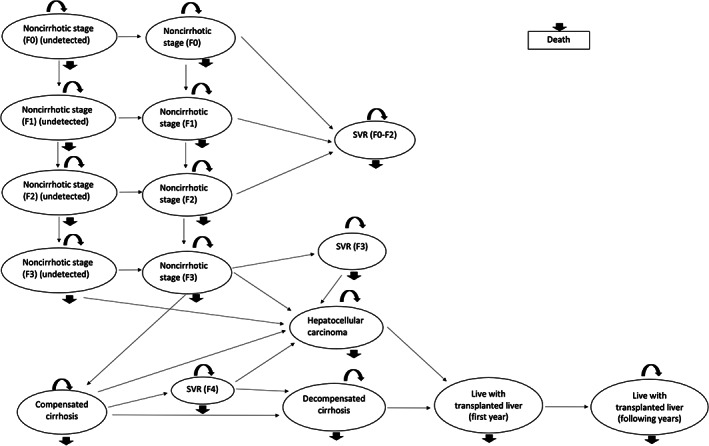
Markov model structure for chronic hepatitis C disease progression. SVR, sustained virological response.

The decision‐analytical model for assessing the cost‐effectiveness of alternative HCV testing strategies consisted of a decision tree model (Figure 1) and a Markov model (Figure 2) implemented in TreeAge Pro 2021 [14]. The decision tree started with one of the four alternative testing strategies. Following each testing strategy, there was a chance that the transfusion recipient was infected by HCV, which was determined by the residual risk estimate from a separate analysis (see Table 1, [6]). Once the recipient developed acute hepatitis C, it was assumed that the condition would either clear spontaneously or progress to chronic hepatitis C. For blood transfusion recipients who have achieved blood safety (i.e., no infection transmitted) or whose acute infection cleared spontaneously, it was assumed that they would survive or die of any causes in the following years. For those who progress to chronic infection, a Markov model was used to simulate the disease progression of chronic hepatitis C. The model consisted of a set of mutually exclusive health states where patients either stayed in their current state or transited to other health states or died. Patients would progress through non‐cirrhotic stages (F0–F3) before developing compensated cirrhosis. While in non‐cirrhotic stages, there was a probability that patients had their chronic hepatitis C detected. It was assumed that all diagnosed HCV infections would be treated by DAA therapy, and there was a high chance of achieving sustained virological response (SVR), whereby HCV is not detected in the blood 12 weeks or more after treatment. Once in the ‘compensated cirrhosis’ state, patients would no longer be able to achieve a full recovery as the liver damage done by cirrhosis could not be reversed. Other advanced disease stages of chronic hepatitis C included decompensated cirrhosis and hepatocellular carcinoma, with another proportion of patients receiving a liver transplant.

**TABLE 1:** Residual risk estimate of hepatitis C virus (HCV) infection following each testing strategy used in the model [6].

Testing strategy^a^	Residual risk
1. Universal testing with anti‐HCV and NAT	1 in 151 million
2. First‐time donor (anti‐HCV and NAT), remaining donor (NAT)	1 in 111 million
3. Transfusible component (anti‐HCV and ID‐NAT), plasma for further manufacture (MP16‐NAT)	1 in 151 million
4. Universal testing with NAT only	1 in 66 million

Abbreviations: ID, individual donor; MP16, minipool 16; NAT, nucleic acid testing.

^a^ For strategies 1, 2 and 4, ID‐NAT is used for transfusible component donations and MP16‐NAT for plasma for further manufacture.

### Model parameters

#### Risk of transfusion‐transmitted HCV infection

In this study, the residual risk refers to the risk that a donation from an HCV‐infected donor is not detected by testing, leading to a transfusion recipient becoming infected with HCV. The residual risk for different testing strategies was estimated based on an in‐house purpose‐built risk model [6]. The more conservative mid‐estimates were used in the baseline analysis (Table 1).

#### Transition probabilities

The proportions of first‐time donations and transfusible component donations were taken from Lifeblood 2020 donation data (Table 2, [6]). The spontaneous viral clearance rate in acute infections was derived from a systematic review where the proportion achieving clearance within 12 months following infection was 0.36 [15]. The transition probabilities associated with chronic hepatitis C disease progression were sourced from previous modelling studies [16, 17, 18, 19]. The mortality rates for non‐cirrhotic stages and compensated cirrhosis were based on the mortality rates of blood transfusion recipients, who have higher mortality than the general population (Table S1). Elevated mortality rates were assigned for ‘decompensated cirrhosis’ and ‘hepatocellular carcinoma’ health states using published estimates [23]. The mortality rates following liver transplant were taken from a project that investigated the future health and economic burden of hepatitis C in Australia [24]. As there is no universal testing for HCV among the general Australian population, we assumed that for non‐cirrhotic stages, only 1% (probability 0.01) of blood transfusion recipients would get tested for HCV each year.

**TABLE 2:** Model parameters.

Model parameters	Value	Reference
Blood donation (year 2020)		Lifeblood internal data
Total number of donations	1,595,364	
First‐time donations	108,544	
Transfusible component donations	774,919	
Number of transfusions (year 2020)	1,194,285	Lifeblood internal data
Transition probabilities (annual)		
Spontaneous clearance	0.3610	[15]
F0–F1	0.1310	[16, 17, 18]
F1–F2	0.0885	[16, 17, 18]
F2–F3	0.1345	[16, 17, 18]
F3 to compensated cirrhosis	0.1235	[16, 17, 18]
F3 to hepatocellular carcinoma	0.0020	[16, 17, 19]
Compensated cirrhosis to decompensated cirrhosis	0.0300	[16, 17, 19]
Compensated cirrhosis to hepatocellular carcinoma	0.0360	[16, 17, 19]
Probability of liver transplant with decompensated cirrhosis	0.0330	[20]
Probability of liver transplant with hepatocellular carcinoma	0.1000	[20]
Mortality following blood transfusion	Age‐specific (Table S1)	[21, 22]
Mortality (decompensated)	0.0390	[23]
Mortality (hepatocellular carcinoma)	0.1760	[23]
Mortality (liver transplant first year)	0.1690	[24]
Mortality (liver transplant following years)	0.0340	[24]
Cost (AUD 2020)		
*HCV testing* (*aggregated cost per donation*)		Lifeblood internal estimate
Anti‐HCV testing	$7.20^a^	
ID‐NAT (transfusible component)	$9.40^a^	
MP16‐NAT (plasma for further manufacture)	$5.30^a^	
*Health state cost*		
F0–F2	$554.85	[20]
F3	$858.11	[20]
Compensated cirrhosis	$1161.43	[20]
Decompensated cirrhosis	$18,883.02	[20]
Hepatocellular carcinoma	$13,364.74	[20]
*Transition cost*		
HCV diagnosis	$ 1163.39	[20]
DAA interferon‐free therapy	$12,661.22	PBS code 11147Q
Post‐treatment for SVR	$377.04	[20]
Transition from F3 to compensated	$702.62	[20]
Transition to HCC	$1205.18	[20]
Liver transplant	$180,807.05	[20]
Utility		
*Health state utility*		
Australian population norms	0.91	[25]
SVR (F0–F2)	0.85	Assumed
SVR (F3)	0.82	Assumed
SVR (F4)	0.79	[26]
Non‐cirrhotic stages (F0–F3)	0.75	[26]
Compensated cirrhosis	0.67	[26]
Decompensated cirrhosis	0.60	[26]
Hepatocellular carcinoma	0.66	[26]
Live with transplanted liver	0.66	[26]

Abbreviations: anti‐HCV, hepatitis C virus antibody; ID, individual donor; MP16, minipool of 16 donations; NAT, nucleic acid testing; SVR, sustained virological response.

^a^ This is an aggregated estimate of test cost per donation for the purposes of the economic analysis including, but not representative of, the cost of consumables procured by Lifeblood.

#### Resource use and costs

This cost‐effectiveness analysis was conducted from a healthcare system perspective where only direct costs of providing testing and treatment of HCV infection were considered. The costs of testing HCV in blood donations were the rolled‐up costs including the costs of tests and labour. As NAT is a multiplex test that also tests for human immunodeficiency virus (HIV) and hepatitis B virus in blood donations, it should be noted that the costs of NAT listed in Table 2 are not just for hepatitis C alone. In the baseline analysis, the total costs of NAT were used as the costs of NAT for HCV. An alternative assumption that the costs of NAT for HCV were a third of the total costs of NAT was tested in the sensitivity analysis. The costs of managing different stages of chronic hepatitis C were sourced from an Australian study that assessed the cost‐effectiveness of treating people who inject drugs with DAA therapy [20]. All cost items were valued in 2020 Australian dollars.

#### Health outcome measure

The health impact of HCV infection was quantified using quality‐adjusted life years (QALYs). QALYs are calculated by multiplying the utility weight associated with a health state by the number of years lived in that state. The utility weights range between 0 and 1, with 0 representing death and 1 representing full health. The utility weight for the Australian general population was used for those blood transfusion recipients who were not infected by HCV [25]. The utility weights for different stages of chronic hepatitis C were informed by a recent systematic review and meta‐analysis of health utilities in patients with chronic hepatitis C, including people who are treated with DAA [26]. As the chronic hepatitis C condition deteriorates, the utility weight would decrease accordingly. Those infected but achieving SVR were assumed to experience improved quality of life, but the utility weight would still be lower than for the general population. We also applied a disutility of 0.1 to the utility weights to all health states to account for the impact of health conditions that required transfusion.

### Model evaluation

#### Baseline analysis

Three age groups were modelled in this study to account for different survival rates following blood transfusion (0–35 years, 36–65 years and 66+). The Markov model was run with an annual cycle length and a time horizon of 50, 30 and 20 years for the three age groups, respectively. The costs and health outcomes (QALYs) were accumulated as the model ran through health states until reaching the end of the time horizon. Both costs and QALYs were discounted at a rate of 5% per year in line with Australian government guidelines [27, 28]. The main outputs from the Markov model were fed into the decision tree model where each pathway was associated with cost and health outcome payoffs. The expected cost and health outcomes of implementing a testing strategy were computed by summing the costs and health outcomes weighted by the probability of each outcome (‘rollback’ analysis). The total testing costs for HCV in blood donations in Australia were also estimated using the actual number of blood donations in 2020 and 2021.

#### Sensitivity analysis

To account for the model parameter uncertainty, we conducted a sensitivity analysis to assess the impact of varying parameter values on model outputs. Moreover, we conducted a threshold analysis to determine the parameter values required for the testing strategies to become cost‐effective.

## RESULTS

### Baseline analysis

The expected costs and QALYs associated with 1000 blood donations under different testing strategies are presented in Table 3. As the residual risks are so low, the number of potential cases of acute hepatitis C and chronic hepatitis C approximates to zero in each scenario. Thus, the costs of managing HCV infections had virtually no impact on the total costs (which were determined by the costs of testing), and the total QALYs contributed by uninfected blood transfusion recipients were the same for all four testing strategies. As a result, universal testing with NAT only is the preferred strategy in our analysis as it had the lowest testing cost for all three age groups.

**TABLE 3:** Expected total costs and quality‐adjusted life years (QALYs) per 1000 blood donations.^a^

Testing strategy^b^	Costs (AUD)	QALYs (0–35 years)	QALYs (36–65 years)	QALYs (66+ years)
1. Universal testing with anti‐HCV and NAT	$14,492	22,817.24	9432.32	4633.82
2. First‐time donor (anti‐HCV and NAT), remaining donor (NAT)	$7781	22,817.24	9432.32	4633.82
3. Transfusible component (anti‐HCV and ID‐NAT), plasma for further manufacture (MP16‐NAT)	$10,789	22,817.24	9432.32	4633.82
4. Universal testing with NAT only	$7292	22,817.24	9432.32	4633.82

Abbreviations: anti‐HCV, hepatitis C virus antibody; ID, individual donor; MP16, minipool 16; NAT, nucleic acid testing.

^a^ Incremental cost‐effectiveness ratios were not reported as the difference in QALYs was negligible.

^b^ For strategies 1, 2 and 4, ID‐NAT is used for transfusible component donations and MP16‐NAT for plasma for further manufacture.

Based on the actual number of blood donations in 2020 and 2021, the costs of testing blood donations for HCV using different strategies were calculated and are presented in Table 4. The costs of dual testing were estimated to be A$23 million in 2020 and increased as the volume of blood donations increased in 2021. If universal testing with NAT only were to be implemented, the total costs of testing would be halved, and the annual cost savings could reach A$11 million.

**TABLE 4:** Number of blood donations and estimated total testing costs for years 2020–2021.

	2020	2021
Number of blood donations		
Total	1,595,364	1,603,507
First‐time	108,544	94,916
Repeat	1,486,820	1,508,591
Transfusible component	774,919	848,637
Plasma for further manufacture	820,445	754,870
Costs (AUD)		
1. Universal testing with anti‐HCV and NAT	$23,119,218	$23,523,249
2. First‐time donor (anti‐HCV and NAT), remaining donor (NAT)	$12,414,114	$12,661,394
3. Transfusible component (anti‐HCV and ID‐NAT), plasma for further manufacture (MP16‐NAT)	$17,212,014	$18,088,185
4. Universal testing with NAT only	$11,632,597	$11,977,999

Abbreviations: anti‐HCV, hepatitis C virus antibody; ID, individual donor; MP16, minipool 16; NAT, nucleic acid testing.

### Sensitivity analysis

Given that the background risks of HCV transmission are extremely low, varying residual risks and changing the value of parameters related to managing HCV infection had virtually no impact on the baseline results. We greatly increased the residual risk of other testing strategies in the threshold analysis. The results show that for the current practice to be cost‐effective, the residual risk of other testing strategies would need to be at least one HCV infection per 2424 donations, which is over 60,000 times the baseline estimate for residual risk (1 in 151 million, Table 1).

## DISCUSSION

To our knowledge, this is the first published study to assess the cost‐effectiveness of removing anti‐HCV blood donation testing. We tested different scenarios where NAT was applied solely for repeat donations, fractionated donations or all blood donations. Given that the residual risks of acquiring HCV following blood transfusion are extremely low for each proposed testing strategy, our modelling predicts that almost no one will develop acute or chronic hepatitis C, incur substantial costs associated with managing hepatitis C and experience reduced quality of life. As a result, the cost‐effectiveness of different testing strategies is almost completely determined by the cost of testing. Therefore, strategy 4 (the NAT‐only testing) is clearly the optimal strategy due to its lower testing cost.

Although we are not aware of published studies assessing the impact of removing anti‐HCV testing from blood donation screening, our finding that a single test is more cost‐effective than dual testing is consistent with previous cost‐effectiveness studies [11, 12, 13]. These studies assessed the cost‐effectiveness of adding NAT to serological (antibody and antigen) testing for blood donations and all reported that adding the additional test would not be cost‐effective. This is because using a serological test alone already reduces residual risks to a very low level. Adding NAT further lowers the residual risk but the additional reduction in viral transmission is minimal, while the additional cost of testing is significant. Similarly in our case, although removing the anti‐HCV test would result in a slightly higher residual risk, the impact on total costs of managing HCV infections is negligible given an already very low background risk. The results from our threshold analysis also showed that the background risk needs to be elevated to a very high level for the dual testing strategy to be cost‐effective. It should be noted that our study did not include a scenario where anti‐HCV alone was used for screening. The residual risk of using anti‐HCV alone is many fold higher at 1 in 800,000, as estimated by the Lifeblood internal modelling. In addition, NAT also tests for hepatitis B and HIV. A strategy with anti‐HCV testing alone was, therefore, not considered a realistic option in the Australian setting. Although our study findings may not be comparable to those reported in the previous cost‐effectiveness studies, this study and previous cost‐effectiveness studies are concordant in determining that a single test would suffice in blood donations, and dual testing is not cost‐effective.

One factor distinguishing this study from earlier cost‐effectiveness analyses is that we assessed targeted testing based on donation collection types. We found that the costs of targeted testing would increase as the proportion of donations receiving dual testing increases. In our case, first‐time donations represented approximately 10% of total donations, so screening first‐time donations with dual testing and repeat donations with NAT only is the second least costly testing strategy. Screening transfusible component donations with dual testing and screening plasma for further manufacture with MP16‐NAT only would cost much more because almost 50% of blood donations are transfusible component donations, although as Australia becomes more plasma focused this proportion will change.

A limitation is the lack of population‐level HCV screening rate data. In Australia, HCV screening is not conducted routinely for blood transfusion recipients nor for the general population, so we conservatively assumed a 1% annual diagnosis of theoretically infected blood transfusion recipients. Although the HCV screening rate would directly impact on the chronic HCV disease progression and associated costs (with earlier detection and treatment preventing severe complications), it does not affect the analysis due to the very small risks of viral transmission.

Cost‐effectiveness analysis is one important assessment in considering blood safety risk management but risk‐based decision‐making includes other assessments, including stakeholder view, reputational risk and ethics [29, 30]. Historically, blood operators have tended to risk‐mitigate at any cost, but with risk‐based decision‐making principles, blood operators are moving to risk reduction. One recent example includes a transfusion‐transmitted hepatitis C case in Germany that occurred with minipool testing that would likely have been prevented by ID‐NAT [31]. Despite this, the authors concluded that it remains a very rare event, and the implication is, therefore, that the risk is considered tolerable. Our cost‐effectiveness analysis clearly concludes that the screening strategy for HCV in blood donations currently implemented in Australia is not cost‐effective compared with targeted testing or universal testing with NAT only. Partial or total removal of anti‐HCV testing would bring significant cost savings without compromising blood supply safety. The purpose of risk management is not to eliminate risk but to use resources appropriately to minimize or accept the risk, and with overwhelming evidence of ineffectual resource use in our case, this provides a strong argument to cease or change anti‐HCV testing.

## CONFLICT OF INTEREST STATEMENT

All authors declare no conflicts of interest.

## Supporting information


**Table S1.** Mortality following blood transfusion by age groups.
