# Supplementary material for: Removing hepatitis C antibody testing for Australian blood donations: A cost‐effectiveness analysis
Source: Vox Sang. 2023 May 14;118(6):471–9. doi: 10.1111/vox.13429 (PMC10952740; doi:10.1111/vox.13429)
Supplement: Supplementary file 1 — Table S1. Mortality following blood transfusion by age groups. [file VOX-118-471-s001.docx]

**Supplementary File**

Table A1. Mortality following blood transfusion by age groups*

| Year | 0 – 35 | 36 – 65 | 66+ |
| --- | --- | --- | --- |
| 1 | 0.1735 | 0.3337 | 0.4190 |
| 2 | 0.0257 | 0.0618 | 0.0776 |
| 3 | 0.0101 | 0.0334 | 0.0582 |
| 5 | 0.0054 | 0.0231 | 0.0427 |
| 10 | 0.0058 | 0.0180 | 0.0344 |
| 11 | 0.0005 | 0.0056 | 0.0618 |
| 12 | 0.0005 | 0.0061 | 0.0666 |
| 13 | 0.0006 | 0.0066 | 0.0718 |
| 14 | 0.0006 | 0.0072 | 0.0772 |
| 15 | 0.0007 | 0.0078 | 0.0833 |
| 16 | 0.0007 | 0.0085 | 0.0898 |
| 17 | 0.0008 | 0.0093 | 0.0971 |
| 18 | 0.0008 | 0.0101 | 0.1050 |
| 19 | 0.0009 | 0.0110 | 0.1139 |
| 20 | 0.0009 | 0.0120 | 0.1237 |
| 21 | 0.0010 | 0.0130 | 0.1346 |
| 22 | 0.0011 | 0.0142 |  |
| 23 | 0.0012 | 0.0155 |  |
| 24 | 0.0013 | 0.0169 |  |
| 25 | 0.0013 | 0.0185 |  |
| 26 | 0.0015 | 0.0202 |  |
| 27 | 0.0016 | 0.0221 |  |
| 28 | 0.0017 | 0.0240 |  |
| 29 | 0.0018 | 0.0261 |  |
| 30 | 0.0020 | 0.0282 |  |
| 31 | 0.0021 | 0.0304 |  |
| 32 | 0.0023 |  |  |
| 33 | 0.0025 |  |  |
| 34 | 0.0027 |  |  |
| 35 | 0.0029 |  |  |
| 36 | 0.0031 |  |  |
| 37 | 0.0034 |  |  |
| 38 | 0.0037 |  |  |
| 39 | 0.0040 |  |  |
| 40 | 0.0044 |  |  |
| 41 | 0.0048 |  |  |
| 42 | 0.0052 |  |  |
| 43 | 0.0056 |  |  |
| 44 | 0.0061 |  |  |
| 45 | 0.0066 |  |  |
| 46 | 0.0071 |  |  |
| 47 | 0.0077 |  |  |
| 48 | 0.0083 |  |  |
| 49 | 0.0090 |  |  |
| 50 | 0.0098 |  |  |
| Source: Year 1 – 10: calculation based on Lifeblood internal data and Borkent-Raven et al.[1];Year 10 – 50: Australian Bureau of Statistics [2]  *Calculations were based on all patients. | | | |

1. Borkent-Raven BA, Janssen MP, van der Poel CL, Schaasberg WP, Bonsel GJ, van Hout BA. Survival after transfusion in the Netherlands. Vox Sang. 2011;100(2):196-203.

2. Australian Bureau of Statistics. Deaths, Year of occurrence, Age at death, Age-specific death rates, Sex, States, Territories and Australia 2022 [14 Nov, 2022]. Available from: <https://www.abs.gov.au/statistics/people/population/deaths-australia/2021>.
